# Blood pressure profile of primary school children in Eastern Cape province, South Africa: prevalence and risk factors

**DOI:** 10.1186/s12887-022-03221-5

**Published:** 2022-04-14

**Authors:** Howard Gomwe, Eunice Seekoe, Philemon Lyoka, Chioneso Show Marange

**Affiliations:** 1 Sefako Makgatho Health Sciences University, Pretoria, South Africa; 2 Department of Nursing Science, Faculty of Health Sciences, University of Fort Hare, East London, South Africa; 3 Department of Statistics, Faculty of Science and Agriculture, University of Fort Hare, East London, South Africa

**Keywords:** Blood pressure, BMI, Children, Overweight, Obesity, Prevalence, South Africa

## Abstract

**Abstract:**

**Background:**

The problem of cardiovascular diseases and lack of adequate information about the blood pressure profiles among children in South Africa has enormous consequences for public health and the general well-being of communities.

**Aim:**

The aim of this study is to determine the blood pressure profiles and associated risk factors of primary school children in South Africa.

**Methods:**

A cross sectional study was conducted among 876 children aged 9 to 14 years from 18 randomly selected schools in the Eastern Cape province of South Africa. Standardised blood pressure measuring instruments were used and an average of three readings was considered. Blood pressure status was classified according to the percentiles of systolic blood pressure (SBP).

**Results:**

The overall prevalence of hypertension was 5.2% and pre-hypertension was 18.5% while normal blood pressure was 76.3%. The multilevel binary logistic regression’s crude and adjusted analysis revealed that increase in age was significantly associated with elevated BP in children ([crude OR = 1.17 [1.05 – 1.29] and [adjusted OR = 1.12 [1.01 – 1.25]). In addition, increase in BMI was significantly associated with elevated BP in children ([crude OR = 1.08 [1.04 – 1.12] and [adjusted OR = 1.06 [1.02 – 1.11]). There was no statistically significant association between elevated BP and gender for both the univariate and multivariate models. There was also no statistical significant risk for elevated BP associated with place of residence.

**Conclusions:**

In this sampled population the established proportion of primary school children with elevated BP is of great concern. In addition, older children and those with high BMI (that is, overweight/obesity) were associated with elevated BP. Interventions towards promoting healthy lifestyles among school learners is a necessity if we are to prevent cardiovascular diseases.

## Background/introduction

Elevated blood pressure has become a common health problem in children. Most people used to think that hypertension is a disease associated only with elderly people while nothing was known about childhood hypertension. The worldwide prevalence of hypertension in teenagers has been reported to be around 1 to 5% and the prevalence in young school learners varies [1, 2]. Research studies on blood pressure in young children give vital information which may assist in identifying and monitoring coronary risk factors [3–5]. Therefore, in order to identify those who are at high risk of developing hypertension later in life, it is essential to screen children for blood pressure at an early stage of their childhood. Studies have shown that over time children with high blood pressure are more likely to be hypertensive when they become adults [6–9]. Early control of hypertension could help to prevent mortality and morbidity caused by high blood pressure. Significant evidence from research studies states that hypertension starts from childhood [10–12]. Conducting blood pressure studies among primary school learners will provide vital data that is helpful in the early identification of risk factors associated with cardiovascular diseases later in life [13].

There are strong associations between being overweight and high blood pressure in children [14–16]. According to [17] the most significant risk factors of hypertension in children and adolescents are overweight and obesity. Liang, et al. [18] reported that children who are overweight and obese have a higher likelihood of developing elevated blood pressure as compared to those with normal weight. It has also been reported that overweight is the main determinant of high blood pressure in children [19]. In a research study conducted by Bhimma et al. [20], findings reveal that the prevalence of high blood pressure was found to increase with an increment in weight as well as factors such as age, height, waist circumference, more fatty substance levels and family background. On the other hand, physical inactivity, which usually results in increased weight and/or body mass index (BMI), has been reported as an associated danger for hypertension [21]. Thus, physical inactivity levels, mostly due to sedentary behaviour, also leads to elevated blood pressure [22]. According to Sorof and Daniels [23] and [24], obesity was one of the risk factors associated with elevated blood pressure in children, including family history of hypertension, dietary and eating habits, and stress levels as well as sedentary behaviours.

Environmental setting is also a contributing factor of hypertension in children [11, 25]. The environment in which children live influences their lifestyle [26, 27]. Kidy, et al. [28] conducted a research study in the rural and urban areas of Uganda to assess the blood pressure profile in primary school children. The results indicated that high blood pressure was associated with residence where more children from urban areas had elevated blood pressure as compared to those from rural areas. In South Africa, studies that were mostly conducted among rural children and adolescents indicated that the prevalence of hypertension ranged from 1 to 25.9% [19, 29]. Contrary to developed environmental settings such as China, Meng, et al. [30] reported that the high blood pressure prevalence in children aged between 3 and 18 years was 3.1%. On the other hand, Lewis, et al. [31] reported that between 2011 and 2014 the prevalence of hypertension in children aged between 8 and 17 years old in China was 2.2%. Several studies showed a high prevalence of overweight/obesity amongst children and adolescents in South Africa and sub-Saharan Africa [32–37].

Some studies have shown that elevated BP is associated with increase in age of children [38, 39]. Bhimma et al. [20] stated that blood pressure in children increased with age and height simultaneously. When children grow, their body weight increases and this affects the arterial walls of the heart due to accumulation of cholesterol in the arteries, which leads to atherosclerosis [20]. In general, when children mature they increase in body size and accumulate body fat, which is detrimental to health [20]. Various studies have reported biological and behavioural differences between boys and girls on hypertension. These differences are particularly evident in adolescence, during which sex hormones are the major contributing factor [40]. Other research studies also show that overweight and obesity amongst children and adolescents are strongly dependent on age, gender and populations [41–43].

Identifying the risk factors of blood pressure in children may help to prevent the risk factors that cause hypertension later in adulthood [44]. Several recommendations on integrating blood pressure measurements of primary schoolchildren in paediatric screening have been made [45]. Nevertheless, such recommendations have been ignored mainly due to the fact that hypertension in children is not considered as severe as that in adults. In spite of the potential benefits, the desire to establish and conduct lifestyle interventions in children is not a priority, but rather most of them target the adult population only [46]. Lifestyle interventions targeting young children should rather be prioritised for prevention of hypertension and other lifestyle related chronic diseases. The prevalence of hypertension among South African primary school children is not known regardless of evidence of increasing childhood obesity [47]. The blood pressure profiling on childhood hypertension is scant and there is lack of consistency in the studies from the African continent. Therefore, it is vitally important to know the blood pressure profile and associated risk factors for high blood pressure in primary school children in South Africa. Identifying the risk factors for high blood pressure profile in children in South Africa will help in prevention management. This study was therefore conducted to assess the blood pressure profile and its associated risk factors among primary school learners in South Africa.

## Methodology/design

The study used a cross-sectional descriptive design with primary school learners in the Eastern Cape province of South Africa. The study involved a random sample of 876 learners aged 9 to 14 years. Standardised blood pressure measuring instruments were used and an average of three readings was considered. Blood pressure status was classified according to the percentiles of systolic blood pressure (SBP).

### Sampling

The research study utilised three municipalities within the Eastern Cape province, namely, Buffalo City Metropolitan (Amathole municipality), Oliver Tambo and Chris Hani municipalities. These three municipalities represent urban and rural communities and were conveniently and purposively selected for the purposes of comparison of the diverse contexts that exist in the targeted geographical space. The district education departments of each selected municipality provided a list of schools from quintiles 1, 2 and 3 on which the random selection of schools was based. The quintile rankings is a system that the South African Department of Education uses to divide all public schools for purposes of allocation of financial resources. In quintile one there are schools which are the poorest, while in quintile five there are schools which are least poor. We considered schools only in quintiles 1, 2 and 3 that are declared to be no-fee paying schools. Overall, 18 primary schools from quintiles 1, 2 and 3 were randomly selected using a computer-generated programme. Thus, six schools were randomly selected from Amathole municipality (six urban schools) whilst 12 schools were randomly selected from Oliver Tambo (six schools) and Chris Hani (six schools) municipalities (rural setting). Lastly, class registers were used to randomly draw a 10th of the population from each randomly selected school. A total of 870 (411 from urban schools and 459 from rural schools) participants were recruited and participated in the study.

### Ethical considerations

The research ethics committee of the hosting university approved the study protocol and issued an ethical clearance (Certificate reference number: REC-270710-028-RA Level 01) as permission for the study to be conducted. In addition, researchers also sought permission to conduct the study from the Research Ethics Committees of the Department of Education as well as the Department of Health in the Eastern Cape Province of South Africa. After obtaining all the necessary ethical clearance letters, the researchers then conducted the data collection process. The scope and nature of the study were explained to the selected primary school learners as well as their parents. Since the study was looking at learners aged 9 to 14 years, parental consent was obtained. Confidentiality and anonymity were maintained as guided by the study protocol.

### Body mass index (BMI) measurement

The height (in cm) and weight (in kg) were measured to determine the BMI. Height and weight were measured with a stadiometer scale with heights. The children were weighed bare footed in order to measure the correct weight. The two measurements were used to determine the BMI. The standardised formula for weight and height (weight/height^2 (in kg/m^2^)) was utilised to determine the BMI. BMI was categorised as underweight, healthy weight, overweight and obese, based on the World Health Organisation (WHO) classification of the BMI standard.

### Blood pressure (BP) measurement

Blood pressure was measured three times on the left upper arm at 5-min interval using an electronic BP monitor. The final BP measurement was recorded as the average of the three measurements. The measurements were done in the morning before break when children were not active. The children were told to relax for a few minutes before measuring blood pressure. The measurements were then classified according to blood pressure percentiles. Thus, normal blood pressure – average systolic blood pressure (SBP) < 90th percentile; pre-hypertension – SBP ≥ 90th percentile but <95th percentile; hypertension – average SBP ≥ 95th percentile. Elevated blood pressure was then defined as the average SBP ≥ 90th percentile, which is the combination of pre-hypertension and hypertension.

### Statistical analysis

All statistical analyses were performed using the Statistical Package for the Social Sciences (SPSS Version 27). Firstly, a descriptive analysis on the demographic and anthropometric characteristics of respondents was conducted using means, standard deviations as well as frequencies and the associated percentages. An independent samples T-Test and the Chi-square test were used to establish the differences and associations that exist within the demographic and anthropometric characteristics with elevated blood pressure. Due to clustering, the multilevel binary logistic regression model was used to generate relative risk estimates (both unadjusted and adjusted with their respective 95% confidence intervals) to assess factors associated with elevated BP. Factors investigated in the univariate and multivariate models included gender (boys and girls), age (in years as a continuous variable), place of residence (rural and urban) and body mass index (in kg/m^2^ as a continuous variable). It is important to note that age and BMI were treated as continuous variables in the multilevel regression analysis.

## Results

Table 1 shows the demographic and anthropometric characteristics of the primary school children who participated in the study. Of the 870 children, the majority were girls (*n* = 519; 59.7%). The sample had a mean age 11.0 ± 1.5 years with most children aged between 11 to 12 years. The majority of these children resided in rural settings (*n* = 459; 52.8%) whilst 411 (47.2%) were residing in urban areas. The mean body mass and height were 39.3 ± 10.4 and 144.1 ± 10.8 respectively. The mean BMI was 18.8 ± 4.1 kg/m^2^, with 3.7% being classified as overweight and 2.2% as obese. On the other hand, the mean of SBP and DBP were 107.8 ± 19.4 and 66.7 ± 13.3 respectively. Overall, the established proportion of primary school children with normal blood pressure was 76.3% whilst 5.2% were hypertensive. We then further classified the blood pressure categories to normal (normal blood pressure) and elevated blood pressure (pre-hypertension and hypertension). From this classification, the prevalence of elevated blood pressure in the sample was 23.7%.

**Table 1:** Demographic and anthropometric characteristics of respondents

Characteristic	N (Valid %) or Mean ± SD
Gender	
*Boys*	351 (40.3)
*Girls*	519 (59.7)
Age (years)	11.0 ± 1.5
Age Categories	
*9 to 10 years*	222 (25.5)
*11 to 12 years*	423 (48.6)
*13 to 14 years*	225 (25.9)
Residence	
*Urban*	411 (47.2)
*Rural*	459 (52.8)
Weight (kg)	39.3 ± 10.4
Height (cm)	144.1 ± 10.8
BMI (kg/m^2^)	18.8 ± 4.1
BMI Categories	
*Underweight*	540 (62.1)
*Normal*	279 (32.1)
*Overweight*	32 (3.7)
*Obese*	19 (2.2)
SBP (mmHg)	107.8 ± 19.4
DBP (mmHg)	66.7 ± 13.3
Blood Pressure Categories	
*Normal Blood Pressure*	664 (76.3)
*Pre-Hypertension*	161 (18.5)
*Hypertension*	45 (5.2)
Elevated Blood Pressure	
*Yes*	206 (23.7)
*No*	664 (76.3)

*N* = 870

In Table 2, we present the results for the comparisons and associations of elevated blood pressure with the demographic and anthropometric characteristics of respondents. In this sample, there existed no statistically significant differences in the proportion of elevated BP by gender (*p =* 0.187). On the other hand, there was a statistically significant association between the proportions of age and BP (*p =* 0.010). Thus, the proportion of elevated BP was lower for 9 to 10 year old children (42/222 = 18.9%) as compared to that of 11 to 12 year olds (95/423 = 22.5%) and that of 13 to 14 year old children (69/225 = 30.7%). In general, the mean age of children with elevated BP (11.3 ± 1.6) was significantly higher (*p =* 0.002) than those with normal BP (10.9 ± 1.5). However, there were no significant associations and differences in proportions in terms of place of residence and whether someone has normal BP or elevated BP. The mean BMI of those with elevated BP (19.9 ± 4.6) was significantly higher than that of children with normal BP (*p* < 0.0001). There was a similar result with weight (*p* < 0.0001) and height (*p =* 0.007). There was also a statistically significant association between BMI and BP. Thus, the proportion of elevated BP was lower for underweight (97/540 = 18.0%) and normal weight children (89/279 = 31.9%) as compared to 43.8% (14/32) among overweight children (*p* < 0.0001). Children with elevated BP had significantly (all *p* < 0.001) higher mean values for SBP (Mean = 107.8; SD = 19.4) and DBP (Mean = 66.7; SD = 13.3).

**Table 2:** Comparisons and associations of demographic and anthropometric characteristics of respondents with elevated blood pressure

Characteristic	All	Normal BP	Elevated BP	*p*-value
	N(%) or Mean ± SD	N(%) or Mean ± SD	N(%) or Mean ± SD	
Gender				
*Boys*	351 (40.3)	276 (41.6)	75 (36.4)	0.187
*Girls*	519 (59.7)	388 (58.4)	131 (63.6)	
Age (years)	11.0 ± 1.5	10.9 ± 1.5	11.3 ± 1.6	0.002*
Age Categories				
*9 to 10 years*	222 (25.5)	180 (27.1)	42 (20.4)	0.010*
*11 to 12 years*	423 (48.6)	328 (49.4)	95 (46.1)	
*13 to 14 years*	225 (25.9)	156 (23.5)	69 (33.5)	
Residence				
*Urban*	411 (47.2)	317 (47.7)	94 (45.6)	0.596
*Rural*	459 (52.8)	347 (52.3)	112 (54.4)	
Weight (kg)	39.3 ± 10.4	38.3 ± 9.7	42.6 ± 11.5	< 0.0001*
Height (cm)	144.1 ± 10.8	143.4 ± 10.2	146.0 ± 12.4	0.007*
BMI (kg/m^2^)	18.8 ± 4.1	18.5 ± 3.9	19.9 ± 4.6	< 0.0001*
BMI Categories				
*Underweight*	540 (62.1)	443 (66.7)	97 (47.1)	< 0.0001*
*Normal*	279 (32.1)	190 (28.6)	89 (43.2)	
*Overweight*	32 (3.7)	18 (2.7)	14 (6.8)	
*Obese*	19 (2.2)	13 (2.0)	6 (2.9)	
SBP (mmHg)	107.8 ± 19.4	100.1 ± 13.5	132.8 ± 13.7	< 0.0001*
DBP (mmHg)	66.7 ± 13.3	64.4 ± 12.5	74.0 ± 13.2	< 0.0001*

(*) Statistically significant differences at Alpha = 0.05

Table 3 presents the crude and adjusted analysis of risk factors for elevated BP. There was no statistically significant association between elevated BP and gender for both the univariate and multivariate models. However, being older was significantly associated with elevated BP in children ([crude OR = 1.17 [1.05 – 1.29] and [adjusted OR = 1.12 [1.01 – 1.25]). This was similar with higher BMI which was significantly associated with elevated BP in children ([crude OR = 1.08 [1.04 – 1.12] and [adjusted OR = 1.06 [1.02 – 1.11]). No statistically significant association was reported between elevated BP with place of residence for both the univariate analysis (*p =* 0.635) and the multivariate model (*p =* 0.555).

**Table 3:** Crude and adjusted multilevel binary logistic regression analyses of gender, age, residence and body mass index with prevalence of hypertension among selected primary school children

Characteristics	Crude OR [95% CI] & Beta Estimates			Adjusted OR [95% CI] & Beta Estimates		
	OR [95% CI]	Beta (SE)	*p*-value	OR [95% CI]	Beta (SE)	*p*-value
Gender						
*Boys*	Reference			Reference		
*Girls*	1.24 [0.90 – 1.72]	0.22 (0.16)	0.185	1.16 [0.84 – 1.60]	0.15 (0.16)	0.354
Age (years)	1.17 [1.05 – 1.29]	0.15 (0.05)	0.003*	1.12 [1.01 – 1.25]	0.12 (0.05)	0.033*
Residence						
*Urban*	Reference			Reference		
*Rural*	1.08 [0.79 – 1.47]	0.07 (0.16)	0.635	1.10 [0.80 – 1.50]	0.09 (0.16)	0.555
BMI (kg/m^2^)	1.08 [1.04 – 1.12]	0.07 (0.02)	< 0.0001*	1.06 [1.02 – 1.11]	0.06 (0.02)	0.002*
Intercept					−3.88 (1.58)	0.014*

(*) Statistically significant at Alpha = 0.05

## Discussion

The aim of this research study was to assess the blood pressure profile and risk factors associated with high blood pressure among primary school children in the Eastern Cape province, South Africa. It is necessary to assess blood pressure in school children whilst they are still young in order to identify those at risk of developing hypertension later in life. Research studies have shown that high levels of blood pressure in children are associated with a likelihood of becoming hypertensive later in life [6, 9–11]. Hypertension does not produce signs and symptoms in early life, thus early diagnosis of hypertension could help to prevent mortality and morbidity related to high blood pressure.

The current research study showed a high prevalence of pre-hypertension and hypertension among primary school learners, similar to several studies in South Africa and sub-Saharan Africa [32–34, 36, 37, 41]. The observed prevalence of pre-hypertension was 18.5 and 5.2% for hypertension while those who reported normal blood pressure constituted 76.3%. The prevalence of hypertension and pre-hypertension reported in this research study was greater than 2.6 and 3.5% respectively as reported by Bhimma, et al. [20]. This high prevalence of hypertension in school learners has been associated with increasing BMI or overweight/obesity. There is a relationship between BP and BMI in children, which features the advancement of metabolic disorder [20]. It is well known that overweight and obese children are more likely to be at higher risk of developing pre-hypertension [8, 9, 48] and more than fourfold increased risk of developing hypertension compared with those with normal weight [18]. Our results confirm the results of Monyeki, et al. [19, 29] who also reported the association between high blood pressure and overweight and obesity in children.

This study revealed no significant association between high blood pressure with residence. The reason could be that South African rural areas are developing rapidly such that the standard of living in terms life styles of school learners is as good as that in urban areas. Thus, the government provides transport and nutrition in all quintile 1, 2 and 3 schools. However, some rural quintile schools do not benefit from the transport provided by the government because of the poor roads in the rural areas. The findings were contrary to Kidy, et al. [28] who indicated that high blood pressure was associated with residence. Most urban learners had elevated blood pressure as compared to rural learners. This was because learners from the urban areas are living a sedentary lifestyle whilst those in the rural areas are living an active lifestyle. In addition, our findings revealed that gender was not a statistically significant risk factor for elevated BP in children. This is contrary to other studies that reported overweight and obesity amongst children as being strongly dependent on gender [22, 41–43].

In this research study, blood pressure was noted to increase with age and BMI (i.e., weight and height). Previous studies have shown that blood pressure increases with age [12, 22, 38, 39, 41–43] because as a child is growing there are some changes that take place in terms of weight and height [30]. Children with high BMI are at risk of elevated blood pressure and this was the most common finding in different research studies carried out in South African schools [49]. This finding was similar to other studies conducted in Nigeria [19]. Thus, the most notable risk factors of hypertension found in this study were increased age and high BMI (overweight and obesity).

## Conclusion

The proportion of children with elevated BP in this population is of great concern. In addition, older children and those with high BMI (overweight/obesity) were associated with an increased risk of exhibiting elevated BP. Interventions towards promoting healthy lifestyles among school learners is a necessity if we are to prevent cardiovascular diseases. These findings advocate that relevant stakeholders should implement targeted theory-based, contextually appropriate health promotion interventions (for example, see [50]), which are aimed at promoting the increase of physical activities and healthy lifestyles in the Eastern Cape province of South Africa.

## Data Availability

The dataset used and/or analysed during the current study is available from the corresponding author on reasonable request.
